# Development of a Community-Sensitive Strategy to Increase Availability of Fresh Fruits and Vegetables in Nashville’s Urban Food Deserts, 2010–2012

**DOI:** 10.5888/pcd10.130008

**Published:** 2013-07-25

**Authors:** Celia Larson, Alisa Haushalter, Tracy Buck, David Campbell, Trevor Henderson, David Schlundt

**Affiliations:** Author Affiliations: Alisa Haushalter, Tracy Buck, David Campbell, Trevor Henderson, Metro Public Health Department, Nashville, Tennessee; David Schlundt, Vanderbilt University, Nashville, Tennessee.

## Abstract

**Background:**

Food deserts, areas that lack full-service grocery stores, may contribute to rising rates of obesity and chronic diseases among low-income and racial/ethnic minority residents. Our corner store project, part of the Centers for Disease Control and Prevention’s Communities Putting Prevention to Work initiative, aimed to increase availability of healthful foods in food deserts in Nashville, Tennessee.

**Community Context:**

We identified 4 food deserts in which most residents are low-income and racially and ethnically diverse. Our objectives were to develop an approach to increase availability of fresh fruits and vegetables, low-fat or nonfat milk, and 100% whole-wheat bread in Nashville’s food deserts and to engage community members to inform our strategy.

**Methods:**

Five corner stores located in food deserts met inclusion criteria for our intervention. We then conducted community listening sessions, proprietor surveys, store audits, and customer-intercept surveys to identify needs, challenges to retailing the products, and potential intervention strategies.

**Outcome:**

Few stores offered fresh fruits, fresh vegetables, low-fat or nonfat milk, or 100% whole-wheat bread, and none stocked items from all 4 categories. Major barriers to retailing healthful options identified by community members are mistrust of store owners, history of poor-quality produce, and limited familiarity with healthful options. Store owners identified neighborhood crime as the major barrier. We used community input to develop strategies.

**Interpretation:**

Engaging community residents and understanding neighborhood context is critical to developing strategies that increase access to healthful foods in corner stores.

## Background

In the United States, people living in geographic areas that have a low density of grocery stores and high density of fast food restaurants have higher rates of obesity (1,2) and chronic diseases (1) and lower rates of fruit and vegetable availability and consumption (1,3–5). Food deserts (ie, areas that lack full-service grocery stores) tend to be populated by low-income and racial/ethnic minorities (1,6). In 2010, the Centers for Disease Control and Prevention (CDC) funded 50 communities for 2 years to implement policy, systems, and environmental interventions in an effort to reduce obesity (7). Efforts to improve healthful food access in food deserts through corner and convenience stores have shown promise in metropolitan areas (8,9). We combined several approaches into a single field trial that included input from stakeholders for strategy development, technical support to store owners, and a community-wide media campaign (8). Nashville’s Communities Putting Prevention to Work (CPPW) intervention aimed to increase availability of fresh fruits and vegetables, low-fat and nonfat milk, and 100% whole-wheat bread in neighborhoods with no grocery stores by developing and implementing a process that is sensitive to community needs and concerns (10). Our outcome of interest was the establishment of a set of methods for developing and implementing a corner store initiative that includes food desert identification, a mechanism for community input, education/technical support, and a community-wide campaign.

## Community Context

### Food deserts

We first determined the need to develop a replicable method to identify Nashville’s food deserts. No universal agreement exists on how to define areas where residents have limited access to healthful foods (1,11,12). There is agreement that food deserts provide poor access to healthful foods and consist largely of low-income residents who face transportation barriers to traveling outside their neighborhoods to find full-service grocery stores (13). We developed a detailed algorithm using geographic information technology for identifying food deserts that takes into consideration the presence of food retailers, access to transportation, and other demographic, social, and population health indicators (Appendix A) (14,15). We identified 4 food deserts in Nashville.

### Demographic profile of targeted neighborhoods

The intervention took place in an area of 15,370 people who live in the census tracts (16) of the 5 targeted stores located in the 4 food deserts. Most (64.4%) residents in this area are African American; in contrast, 27.7% of Davidson County’s residents are African American (Table 1). The Nashville 2010–2011 CPPW Behavioral Health Risk Factor Survey documented the following health disparities: 41.1% (standard deviation [SD]), 5.3%) of African Americans were obese, compared to 22.3% (SD, 2.6%) of whites, and 19.6% (SD, 4.2%) of African Americans reported diabetes, compared to 13.3% (SD, 4.2%) of whites. In addition, 55.8% (SD, 3.1%) of whites strongly agreed that it is easy to buy healthful food in one’s neighborhood, compared to 38.4% (SD, 5.2%) of African Americans (17).

**Table 1:** Demographic Characteristics in Targeted Census Tracts Located in Food Deserts, Compared With Nashville/Davidson County and State of Tennessee, 2010

Characteristic^a^	Food Desert 1, Store A	Food Desert 2, Store B	Food Desert 3, Store C	Food Desert 4, Store D	Food Desert 4, Store E	Total Population of All Food-Desert Census Tracts	Davidson County	Tennessee
Census tract population, n	1,816	2,189	5,317	2,047	4,001	15,370	626,681	6,346,105
Female head of household, %	33.5	24.0	25.7	26.8	51.4	44.8	14.7	13.9
Individuals living below federal poverty guidelines, %	31.4	45.7	35.4	42.6	73.7	45.6	17.7	16.9
Households that received Supplemental Security Income or public assistance, or participated in SNAP in previous 12 months	59.9	83.8	63.1	52.6	92.3	72.6	19.5	22.3
Aged 0–17 y, %	27.1	27.0	27.3	25.5	35.6	30.2	21.8	23.5
Aged ≥65 y, %	6.1	8.65	7.9	7.6	3.2	7.5	10.4	13.4
Black/African American, %	91.1	60.3	43.3	51.1	89.6	64.4	27.7	16.7
Hispanic/Latino, %	1.5	1.0	18.9	9.1	3.5	9.1	9.8	4.6
White, %	5.1	35.8	38.4	38.9	5.4	25.4	61.4	77.6

Abbreviation: SNAP, Supplemental Nutrition Assistance Program.

a Source: US Census Bureau (16).

## Methods

Nashville’s CPPW initiative was funded for 2.5 years, from March 2010 through December 2012. During the first year we established partnership contracts, hired and trained staff, developed methodology, and conducted the baseline assessments. The second year focused on refining the implementation and conducting the postassessment. To assist with the development of the corner store initiative, a partnership was established with a local nonprofit community organization, Community Food Advocates, which previously had conducted a formative assessment that identified areas that lacked healthful food resources (18).

### Identification of corner stores

We identified 29 corner stores in the 4 food deserts. We contacted each proprietor by telephone and later conducted an interview at the store. Proprietors were asked to sign an agreement to accept technical assistance; stock the fruits, vegetables, low-fat and nonfat milk, and 100% whole-wheat bread; allow a store audit and customer-intercept interviews; and feature a logo (“So Fresh”) in the store. Our team conducted an observational audit of each store’s surroundings, identifying and recording visible community assets or resources (eg, housing, schools, day-care centers, churches, parks) that would reasonably predict potential success to influence the likelihood of sustainable consumer purchasing. The eligibility criteria for store selection included the following: 1) the store was not exclusively a tobacco and beer or alcohol outlet, 2) the proprietor had an interest in becoming a part of the CPPW intervention and was willing to sign a commitment agreement for the duration of the grant, and 3) the surrounding area included residential or public housing, likelihood of foot traffic or walking distance to schools, child-care facilities, parks, or churches. This process resulted in the identification of 5 stores with at least 1 store in each food desert.

### Baseline assessments

Before data collection, the intervention protocol and survey instruments were submitted and approved by the Metro Public Health Department institutional review board. We then conducted proprietor surveys, store audits, and customer-intercept surveys.

To inform the strategies, we conducted semistructured interviews with proprietors at the store sites. Interview questions sought to gain perceptions of the strengths and challenges associated with being a food retailer in the neighborhood. Sample questions included, “What do you believe are the benefits of having a store at this location?”; “What do you believe are the downfalls?”; “When it comes to the neighborhood, how do the people living around here feel about the store?”; and “How does the store support the community?” A CPPW contract staff member with a background in corner store, retail-food environments conducted the interviews.

We audited the stores for the presence of each of the targeted products using Nutrition Environment Measures Survey–Corner Stores (NEMS–CS), the corner store version of the NEMS–S (grocery store) audit tool and training materials (19) developed by the Philadelphia Food Trust and the Philadelphia Department of Health and used by the Department’s permission. The type, number, and price of each of the following categories of items present in the store were recorded: bread, milk, fresh fruits, and fresh vegetables. Tennessee State University Master of Public Health students conducted the audits and received NEMS–S on-line training (20) and standardized group training.

Intercept surveys were conducted with adult customers at each corner store Monday through Friday, 8:00 AM through 4:30 PM. We used standardized NEMS protocol and training materials. The survey (Appendix B) asked the number and type of food and beverage items purchased. Interviewers were stationed outside the store, and customers were invited to respond to the survey. The eligibility criterion was evidence of a food or beverage purchase. CPPW staff conducted the interviews and received cultural diversity training prior to NEMS training and field work.

### Community engagement for strategy development

We held 2 informal listening sessions to gather information on community stakeholders’ thoughts, ideas, and attitudes. Our intention was to listen to concerns so that we could be alerted to potential barriers and strengthen our strategies as we developed the intervention. A local minister whose church is located in a targeted neighborhood and who is known for being a champion for health equity volunteered to host both sessions and assist with recruitment. Because the study area has many churches, and to inform our strategies from the perspective of those who serve individuals and families, we held 1 listening session with church leaders and clergy. The host minister personally invited church leaders located in or near the target area; 12 African American ministers attended the session. The second listening session was with racially/ethnically diverse community residents and stakeholders, including representatives of housing, food, and social services organizations. The host minister and representatives from neighborhood organizations located in each of the food deserts personally invited residents and stakeholders to the session. At both listening sessions, we described the purpose of CPPW and the corner store intervention and then facilitated discussion. Participants were asked to identify barriers to project success and potential solutions. Following the discussion, participants were served a small meal, of which some items were purchased from a neighborhood corner store by the host minister. No monetary compensation was provided to the participants or the host. Listening sessions were audiotaped and transcribed. We used Atlas-ti 6.2 (ATLAS.ti Scientific Software Development GmbH, Berlin, Germany) to analyze the transcripts and identify common and divergent themes. In addition, a brand and logo, “So Fresh,” developed by the Nashville CPPW media specialist, was presented to the community members to illustrate how it would appear on storefronts. We asked participants to voice their opinions and suggest changes (Figure).

**Figure:**
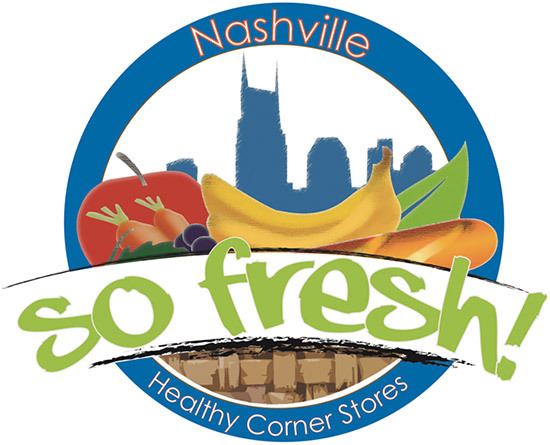
Brand and logo for the Communities Putting Prevention to Work corner store intervention in Nashville, Tennessee, 2010–2012.

## Outcome

### Proprietor interviews

Proprietors stated neighborhood crime or shoplifting or both are the greatest challenges for retailers in their neighborhoods (Table 2). Close proximity to densely populated areas was mentioned as the greatest benefit. Additional benefits named were families as the primary customers and being the sole retailer in the neighborhood. In response to a question about the contribution the store makes to the neighborhood, most owners stated that their customers perceived the store positively. To illustrate investment in the neighborhood, 2 owners reported they participated as a sponsor of community events such as youth sporting events. All store owners reported participating in Supplemental Nutrition Assistance Program (SNAP).

**Table 2:** Proprietor Perceptions, Store Audits, and Customer-Intercept Data From 5 Food-Desert Corner Stores in Nashville, Tennessee, 2011

Survey Item	Food Desert 1, Store A	Food Desert 2, Store B	Food Desert 3, Store C	Food Desert 4, Store D	Food Desert 4, Store E
**Proprietor Perceptions**					
**Barriers to retailing targeted items**					
Neighborhood crime/shoplifting	✓	✓	✓	✓	✓
Lack of structural support for selling items (coolers/displays)		✓			
**Strengths to retailing targeted items**					
Dense residential area	✓		✓	✓	✓
High levels of foot traffic			✓		✓
Families are primary customers		✓			✓
Only store in area/great location		✓		✓	
**Role of store in neighborhood**					
Supports community as a sponsor of sports or events	✓	✓			
Customers proud of or like having store in neighborhood	✓	✓	✓	✓	
Estimated use of SNAP/EBT, %	75	30	40	40	15
**Store Audits**					
**Presence of targeted items stocked**					
Fresh fruit and vegetables			✓	✓	✓
Low-fat or nonfat milk	✓	✓			
100% whole-wheat bread		✓	✓		✓
**Customer-Intercept Data**					
Customer intercepts, n	54	41	32	30	47
Total items purchased, n	78	84	44	53	80
Fresh fruits and vegetables purchased, n	6	5	0	0	0
Low-fat or nonfat milk purchased, n	0	0	0	0	0
100% Whole-wheat bread purchased, n	0	0	0	0	0

Abbreviations: SNAP, Supplemental Nutrition Assistance Program, EBT, electronic benefit transfer.

### Store audits

Two stores stocked low-fat or non-fat milk, 3 stores stocked 100% whole-wheat bread, 3 stores stocked fresh fruit of any kind, and 2 stores stocked vegetables of any kind. Only 1 store stocked foods from only 1 targeted category. No store stocked items from all 4 targeted healthful food categories (Table 2).

### Customer-intercept surveys

From the sample of 204 customer intercepts, we found no purchases of low-fat milk, nonfat milk, or 100% whole-wheat bread and few purchases of fresh fruits or vegetables (Table 3). We were confident the majority of customers who made food and beverage purchases represented the neighborhood because most walked to the stores, and parking was limited.

**Table 3:** Stakeholders’ Perceptions of Barriers to Selling Fresh Produce and Other Healthful Foods and Beverages, Nashville, Tennessee, 2011

Themes	Residents	Clergy
**Consumer education**	“Even the people selling these items . . . aren’t quite sure what they are or how to prepare them.”	“There are . . . people who don’t know the benefits of eating fresh fruits and vegetables.”
	“People would love to eat better, but they don’t know how, or even why they should.”	“There are . . . people who don’t know how to tell if what they’re buying is fresh.”
**Poor-quality produce**	“The quality is not as good as you would find in an actual grocery store. This can cause people to feel like they have to use a lot of canned or frozen goods.”	“When they do have fruits and vegetables, they are too often of such poor quality that we wouldn’t even want to buy them.”
		“Companies vary their quality from store to store in different areas: low-income areas equal worse quality equal higher prices.”
**Mistrust of Store owners**	“Corner stores are not owned by people who have any… connection to the community (don’t live there, didn’t grow up there, didn’t go to school there, don’t go to church there, etc.). Ninety-five percent of . . . residents are African American, but 90% of the businesses . . . are owned by people who are not.”	“Systematic racism in economics: there is no investment in low-income areas, simply because businesses don’t feel they can be profitable.”
	“[Store owners’] perceptions of what people want; eg, ‘I know these people don’t want this, so I’m not going to order it.’”	“This is an overwhelmingly predominantly black community, but there are hardly any store owners who have any kind of connection to the community.”
**Mistrust of government**	“Community residents cringe when they see the government supporting these store owners who don’t necessarily have their best interests at heart.”	“Some people [feel] it is wrong for government to empower stores that take advantage of our communities. . . . We should get some help to build our own.”
	“There is a feeling of resentment about . . . stores receiving incentives . . . keeping up the bad practices of poor customer service and selling goods of inferior quality and for higher prices.”	
	“What happens when the grant ends?”	

### Listening sessions with community stakeholders

Barriers to retailing fresh produce and other healthful options identified by both groups included the need for consumer education, neighborhood history of poor-quality produce offered in small stores, mistrust of store proprietors, and mistrust of government (Table 3). The groups discussed potential solutions to the barriers. Community members suggested that taste tests, free samples, cooking demonstrations, and cooking classes would increase knowledge of fruits and vegetables and how to prepare them. Both groups recommended solutions that address poor-quality produce, such as having mobile farmers markets or setting up fruit and vegetable stands near the stores. Clergy recommended involvement by religious institutions so that partnerships formed among markets, government, and academic institutions could address education, accessibility, and sustainability after the CPPW grant. These types of actions could serve to mitigate and reverse any mistrust that exists between consumers, corner store management, and government agencies. Both groups expressed positive opinions about the logo and brand. Comments indicated that the colors and the image would convey an invitation to customers to purchase the healthful products. We did not analyze the comments by neighborhood or food desert because of the commitment to anonymity of listening session participants.

### Store strategy development and implementation

The approach to strategy development and implementation involved 3 components, which were offered to stores according to their expressed need: 1) technical assistance, 2) partnership development, and 3) a communications campaign to increase awareness of the corner store’s new product offerings. A CPPW corner store team was formed; it comprised several staff members who had previously worked in the study neighborhoods and a contract staff member who had previously managed a similar corner store close to one of the food deserts.

#### Technical assistance

The CDC offered technical assistance to CPPW communities. The CPPW corner store team requested and received education and resources on food procurement and marketing from the National Food Security Council. The team provided education to corner store proprietors, which included consultation on store design and layout, education on methods to assure cleanliness, and information on how to promote purchase of featured items. Additional support included 1) funding for purchase of food displays and coolers, 2) education on where and how to procure products not currently retailed, and 3) advisement on how to promote and direct consumers to targeted items.

The corner store team met with each store owner to review the layout and merchandising of products, suggest alternatives, and identify needs for stocking healthful items. These needs included shelf space for bread and refrigeration space for adding or increasing availability of fresh produce and low-fat or nonfat milk. Two stores received refrigeration units, and 2 stores received display units. We assisted in establishing a relationship between the store proprietors and a mobile market for purchasing seasonal fresh fruits and vegetables. The mobile market made available bulk product packages of various produce items at a lower cost based on procurement by all 5 stores. The 5 stores already had established vendor relationships for purchasing bread and milk.

The corner store team assisted with product placement to increase visibility of healthful items by replacing unhealthful food items or nonfood items with the new, more healthful food options. In addition, colorful signs that displayed the logo were placed near the items. Finally, the team provided samples of foods and beverages made with various fruits and vegetables sold in the store.

#### Partnership development

We served as a liaison to establish relationships between the leaders of churches, community organizations, and corner store proprietors in each targeted neighborhood. The partnership discussions involved developing and implementing a plan so that a neighborhood organization, such as a church, could “adopt” a corner store for purchasing the targeted items.

#### Communications campaign

A 3-tiered media and communications campaign was launched to increase awareness of the corner store initiative among individual consumers, the neighborhood, and the broader community. To increase awareness at the individual level, the corner store brand and logo, “So Fresh,” was printed on promotional posters, signage, and point-of-purchase flags. We assisted store owners with promotional display placement. The neighborhood strategies included installing branded posters outside each store, visible from the sidewalks and street. Announcements of the corner stores’ new product launch were made at neighborhood events and posted on bulletin boards and websites. The minister champion was instrumental in the word-of-mouth campaign across the network of churches. At the community level, the corner store initiative was featured as a component of the larger CPPW community campaign: “NashVitality — the spirit of a healthy, active, and green city.” Billboards that featured the availability of fresh fruits and vegetables in corner stores were placed at strategic locations near the food deserts. Similar advertisements were printed in free publications such as neighborhood magazines and flyers. The corner store initiative was also featured on the CPPW NashVitality social network and Internet campaign.

As a result of attention jurisdiction-wide, opportunities to strengthen the effort emerged from nontraditional partners. For example, students from a private school “adopted” one of the stores and provided cleanup and painting of both the interior and exterior. This type of volunteerism illustrates how unanticipated partnerships may emerge from an effective communications campaign that can result in technical assistance, education about the problem and solutions, and potential sustainable relationships within the larger community.

## Interpretation

The CPPW Nashville corner store initiative is the first field trial in the city to develop and implement an initiative to increase the availability of fresh fruits and vegetables, 100% whole-wheat bread and low-fat or nonfat milk in low-income neighborhood corner stores. We developed an approach informed by community context and community members’ knowledge, attitudes, and experiences. Our experiences during the first year of development and initial stages of implementation yielded many insights and lessons learned.

The baseline results illustrated opportunities to increase access to healthful foods in corner stores. Technical assistance benefited stores in the areas of food procurement, displays, and marketing. Other venues suggested by the community should be considered to increase exposure, availability, and access: mobile farmers markets, community gardens, food tastings, and educational cooking demonstrations.

The greatest challenge for communities is the concern for safety from crime in and around the corner stores. Incorporating police department representatives in strategy development will be an important step to solving this problem. An additional challenge is the lack of trust between the corner store owners and community members. The development of partnerships between stores and community organizations may help to sustain the viability and availability of healthful foods in corner stores by engaging organizations to promote the purchase of targeted items. Relationship building among residents and store owners can serve to mitigate the mistrust that may result from ethnic/racial discordance or cultural differences. Developing community forums such as town hall meetings and listening sessions for dialogue and relationship building can lead to overturning the mistrust between community members, store owners, and government agencies. These opportunities for dialogue can yield information to guide community campaigns including media and strategy development. In addition, although we did not include an evaluation of partnership building and maintenance, a process evaluation can serve to document and inform the effectiveness of such strategies.

Our aim to increase the supply of healthful foods in Nashville’s food deserts yielded a comprehensive, replicable approach, sensitive to community interests and needs. Postevaluation is currently under way. Since baseline data collection, 2 stores have closed; one because of economic downturn and the other because of family illness. From among 3 remaining stores, preliminary evidence suggests an increase in availability of the variety of fruits and vegetables. Efforts will continue to assess the impact, assure sustainability, and promote dialogue with the community to explore additional ways to increase equitable access to healthful foods.

## Appendix A. Methodology for Determining Food Deserts in Nashville, Tennessee

The Nashville Communities Putting Prevention to Work initiative used multiple data sources and a detailed algorithm for identifying food deserts. Data sources included parcel information from Metro City Planning Department, metro tax records identifying food retailers, public transit routes and bus stops from Metro Transit Authority, and population social, economic, and demographic estimates using 2000 Census and Claritas, 2009 census estimates (14) and health status information using Nashville’s REACH 2010 Survey data (15). We identified all full-service grocery stores based on pre-existing knowledge by using the business name and owner such as Walmart, Kroger, Publix, and Piggly Wiggly. The address of each grocery store and all bus routes and bus stops in Davidson County were mapped using ArcView Geographic Information Systems software (Esri, Redlands, California). Aggregated geographic information data was associated with each residential parcel using geocoded location, census tract, or census block group. A residential parcel is a single property identified as having residential use such as a home, trailer, condominium, or apartment building. Distance in miles from each residential household (owner occupied or rented) to the nearest full-service grocery store and metro bus stop were calculated. Thirty-five variables associated with census tracts and census block groups were assembled from existing data sets including the following: the 2000 census, the 2009 Claritas estimates, and random citywide surveys of the Nashville REACH 2010 project (15). These variables reflected demographic characteristics, poverty and social distress, lack of access to transportation, and the prevalence of obesity, diabetes, and hypertension (Table).

Each variable was converted to a *z* score (subtracting the mean and dividing by the standard deviation) across the geographic units (tract or block group). For each household, a weighted sum was computed with *z* score–distance to the nearest grocery given a weight of 5, a *z* score–distance to the bus stop a weight of 2, and the other standardized variables a weight of 1. Higher weights were chosen for household distance to stores and bus stops because these constructs are the most important measures of access to food without transportation at the household level. The use of standardized scores converted all measures to a single metric, which allowed for equal weighting of the economic, demographic, and social factors associated with food deserts.

Mean food desert scores were computed for each census block, as a weighted sum of the household *z* scores, resulting in the scoring of 460 census blocks where food desert scores ranged from −37 to 60. A cutoff score of 20, 1 standard deviation above the mean, was used to identify food deserts.

**Table:** Variables Used to Create Food Desert Index for Nashville, Tennessee

Variable	Description	Sources	Geographic Resolution
**Distance to major grocery stores **	Computed distance from each residential parcel to the nearest grocery store	Parcels and grocery stores (derived from tax records)	Parcel level
**Distance to nearest bus stop **	Computed distance from each residential parcel to the nearest bus stop	Parcels and metro transit	Parcel level
**Elite-impoverished composite **	Factor analytically derived index from census data for Nashville	2000 US census	Block group level
**Comfortable-distressed **	Factor analytically derived index from census data for Nashville	2000 US census	Block group level
**Population mean **	Number of people living in census block group	2009 US census estimates from Claritas	Block group level
**Population density **	People per square mile	2009 US census estimates from Claritas	Block group level
**Percentage white **	Demographics	2009 US census estimates from Claritas	Block group level
**Percentage black **	Demographics	2009 US census estimates from Claritas	Block group level
**Percentage Hispanic **	Demographics	2009 US census estimates from Claritas	Block group level
**Per capita income **	Demographics	2009 US census estimates from Claritas	Block group level
**Median age **	Demographics	2009 US census estimates from Claritas	Block group level
**Percentage married **	Demographics	2009 US census estimates from Claritas	Block group level
**Percentage households occupied by renters **	Housing	2009 US census estimates from Claritas	Block group level
**Percentage households with no car **	Housing	2009 US census estimates from Claritas	Block group level
**Percentage of households with one car **	Housing	2009 US census estimates from Claritas	Block group level
**Average cars per household **	Housing	2009 US census estimates from Claritas	Block group level
**Median household income **	Demographics	2009 US census	Block group level
**Median income whites **	Demographics	2009 US census estimates from Claritas	Block group level
**Median income blacks **	Demographics	2009 US census estimates from Claritas	Block group level
**Percentage children under poverty line **	Demographics	2009 US census estimates from Claritas	Block group level
**Percentage or workforce unemployed **	Demographics	2009 US census estimates from Claritas	Block group level
**Percentage divorced **	Demographics	2009 US census estimates from Claritas	Block group level
**Percentage single **	Demographics	2009 US census estimates from Claritas	Block group level
**Average commute time **	Housing	2009 US census estimates from Claritas	Block group level
**Median housing value **	Housing	2009 US census estimates from Claritas	Block group level
**Percentage of housing that is trailers **	Housing	2009 US census estimates from Claritas	Block group level
**Percentage high school dropouts **	Demographics	2009 US census estimates from Claritas	Block group level
**Percentage of adults below poverty level **	Demographics	2009 US census estimates from Claritas	Block group level
**Percentage of adults who are college graduates **	Demographics	2009 US census estimates from Claritas	Block group level
**Population change from 2000–2009 **	Demographics	2009 US census estimates from Claritas 2000 US census data	Block group level
**Percentage uninsured **	Percentage of people surveyed who reported having no insurance	2001–2004 REACH 2010 data	Census tract level
**Percentage obese **	Percentage of people surveyed reporting BMI ≥30	2001–2004 REACH 2010 data	Census tract level
**Percentage hypertensive **	Percentage of people surveyed reporting a diagnosis of high blood pressure	2001–2004 REACH 2010 data	Census tract level
**Percentage high cholesterol **	Percentage of people surveyed reporting a diagnosis of high cholesterol	2001–2004 REACH 2010 data	Census tract level
**Percentage diabetes **	Percentage of people surveyed reporting a diagnosis of diabetes	2001–2004 REACH 2010 data	Census tract level

## Appendix B. Intercept Survey

Corner store:_____________ Date: ____/____/____ Time: _____:_____

[Baseline /Post] Interviewer Initials: _________

Day of the week: M T W R F

**Table:**

Key Product Information Categories: [B: Beverage] [C: Candy and fruit snacks] [CH: Chips, Pretzels, Popcorn & Crackers] [F: Fruit] [IC: Frozen Treats] [P: Pastry] [PF: Prepared Food] [NSG: Nuts, Seeds & Granola] [O: Other]						
#	Quantity	Size (oz)	Product Brand	Product Name	Product Flavor	Product Category
ex.	2	.067	Frito Lay	Nacho Cheese Doritos	Nacho	CH
1						
2						
3						
4						
5						

Age: [child: 5–12 years] [adolescent: 13–18 years] [adult: 19 or older]
